# Supplementary material for: P-TRAP: a Panicle Trait Phenotyping tool
Source: BMC Plant Biol. 2013 Aug 29;13:122. doi: 10.1186/1471-2229-13-122 (PMC3848748; doi:10.1186/1471-2229-13-122)
Supplement: Additional file 7 — P-TRAP source code. The Netbeans project that contains the source code of the application. [file 1471-2229-13-122-S7.zip › javadoc.zip!/javadoc/org-basmati-workspace/org/basmati/workspace/package-tree.html]

org.basmati.workspace Class Hierarchy (WorkSpace)


JavaScript is disabled on your browser.


- Package
- Class
- Tree
- Deprecated
- Index
- Help

*org.basmati.workspace 1.0*

- Prev
- Next

- Frames
- No Frames

- All Classes

# Hierarchy For Package org.basmati.workspace

## Class Hierarchy

- java.lang.Object
  - org.basmati.workspace.CollectMasterReportAction (implements java.awt.event.ActionListener)
  - java.awt.Component (implements java.awt.image.ImageObserver, java.awt.MenuContainer, java.io.Serializable)
    - java.awt.Container
      - javax.swing.JComponent (implements java.io.Serializable)
        - javax.swing.JPanel (implements javax.accessibility.Accessible)
          - org.basmati.workspace.ScaleSelectorPanel
          - org.basmati.workspace.ScalingPanel
        - org.openide.windows.TopComponent (implements javax.accessibility.Accessible, java.io.Externalizable, org.openide.util.HelpCtx.Provider, org.openide.util.Lookup.Provider)
          - org.basmati.workspace.GraphEditorTopComponent
          - org.basmati.workspace.ParticlesEditorTopComponent
          - org.basmati.workspace.ResultsEditorTopComponent
  - org.openide.loaders.DataObject (implements org.openide.util.HelpCtx.Provider, org.openide.util.Lookup.Provider, org.openide.nodes.Node.Cookie, java.io.Serializable)
    - org.openide.loaders.MultiDataObject
      - org.basmati.workspace.EditandViewGrainFileActionDataObject
      - org.basmati.workspace.ProcessedRiceImageFileDataObject
  - org.basmati.workspace.Drawer
  - org.basmati.workspace.EditResultFileAction (implements java.awt.event.ActionListener)
  - org.basmati.workspace.EditViewGrains (implements java.awt.event.ActionListener)
  - org.basmati.workspace.ExpressRunAction (implements java.awt.event.ActionListener)
  - org.basmati.workspace.FindGrainsAction (implements java.awt.event.ActionListener)
  - org.basmati.workspace.FindGrainsSingleImageAction (implements java.awt.event.ActionListener)
  - org.basmati.workspace.FindGrainTraitsAction (implements java.awt.event.ActionListener)
  - org.basmati.workspace.FindGrainTraitsSingleImage (implements java.awt.event.ActionListener)
  - org.basmati.workspace.FindSkeletonAction (implements java.awt.event.ActionListener)
  - org.basmati.workspace.FindStructureSingleImageAction (implements java.awt.event.ActionListener)
  - org.basmati.workspace.GrainsFinder
  - org.basmati.workspace.GrainTraitsFinder
  - org.basmati.workspace.ImageScaler
  - org.basmati.workspace.ImportImages (implements java.awt.event.ActionListener)
  - org.basmati.workspace.OpenImageRice (implements java.awt.event.ActionListener)
  - org.basmati.workspace.ProjectUIManager
  - org.basmati.workspace.ReportGenerator
  - org.basmati.workspace.ResultProducer
  - org.basmati.workspace.ScaleImage (implements java.awt.event.ActionListener)
  - org.openide.util.SharedClassObject (implements java.io.Externalizable)
    - org.openide.util.actions.SystemAction (implements javax.swing.Action, org.openide.util.HelpCtx.Provider)
      - org.openide.util.actions.BooleanStateAction (implements org.openide.util.actions.Presenter.Menu, org.openide.util.actions.Presenter.Popup, org.openide.util.actions.Presenter.Toolbar)
        - org.basmati.workspace.ColoredImageAction (implements java.awt.event.ActionListener)
        - org.basmati.workspace.ImageTypeAction (implements java.awt.event.ActionListener)
        - org.basmati.workspace.VeiwParticlesAnchors (implements java.awt.event.ActionListener)
  - org.basmati.workspace.StructureFinder
  - org.netbeans.api.visual.widget.Widget (implements javax.accessibility.Accessible, org.openide.util.Lookup.Provider)
    - org.netbeans.api.visual.widget.Scene
      - org.basmati.workspace.ImageViewScene
      - org.netbeans.api.visual.model.ObjectScene
        - org.netbeans.api.visual.graph.GraphScene<N,E>
          - org.basmati.workspace.GraphUI
          - org.basmati.workspace.ParticleScene

## Enum Hierarchy

- java.lang.Object
  - java.lang.Enum<E> (implements java.lang.Comparable<T>, java.io.Serializable)
    - org.basmati.workspace.GraphUI.calcType

- Package
- Class
- Tree
- Deprecated
- Index
- Help

*org.basmati.workspace 1.0*

- Prev
- Next

- Frames
- No Frames

- All Classes
